# Rate of Advancement of Detection Limits in Mass Spectrometry: Is there a Moore’s Law of Mass Spec?

**DOI:** 10.5702/massspectrometry.A0118

**Published:** 2023-04-06

**Authors:** Mark Beattie, Oliver A. H. Jones

**Affiliations:** 1 Australian Centre for Research on Separation Science (ACROSS), School of Science, RMIT University, GPO Box 2476, Melbourne, VIC 3001, Australia

**Keywords:** detection limits, dichlorodiphenyltrichloroethane, signal to noise, glycine, industry

## Abstract

Mass spectrometry is a well-established analytical technique for studying the masses of atoms, molecules, or fragments of molecules. One of the key metrics of mass spectrometers is the limit of detection *e.g.*, the minimum amount of signal from an analyte that can be reliably distinguished from noise. Detection limits have improved greatly over the last 30–40 years to the point that nanogram per litre and even picogram per litre detections are commonly reported. There is however, a difference between detection limits obtained with a single, pure compound in a pure solvent and those obtained from real life samples/matrixes. Determining a practical detection limit for mass spectrometry is difficult because it depends on multiple factors, such as the compound under test, the matrix, data processing methods and spectrometer type. Here we show the improvements in reported limits of detection on mass spectrometers over time using industry and literature data. The limit of detection for glycine and dichlorodiphenyltrichloroethane were taken from multiple published articles spanning a period of 45 years. The limits of detection were plotted against the article’s year of publication to assess whether the trend in improvement in sensitivity resembles Moore’s Law of computing (essentially doubling every two years). The results show that advancements in detection limits in mass spectrometry are close to, but not quite at a rate equivalent to Moore’s Law and the improvements in detection limits reported from industry seem to be greater than those reported in the academic literature.

## INTRODUCTION

Mass spectrometry (MS) is an analytical method used to measure the mass to charge ratio (*m*/*z*) of ions. This data can then be used to the calculate molecular weight and relative abundance of compounds in a sample. Every mass spectrometer consists of three main components. These are, an ionisation source to convert molecules into gas-phase ions; a mass analyser which separates the ions by their *m*/*z* values *via* electric or magnetic fields, and an ion detection system which generates data on the relative abundance of each species with a particular *m*/*z* value.^1)^ Post-acquisition data processing is also an increasingly important part of mass spectrometry. There is considerable variation in all these components, leading to large variation in the types of acquisition, ionisation, fragmentation, mass selection and detection and signal processing in modern mass spectrometers, but the fundamentals are the same across instruments.

**Figure 1:**
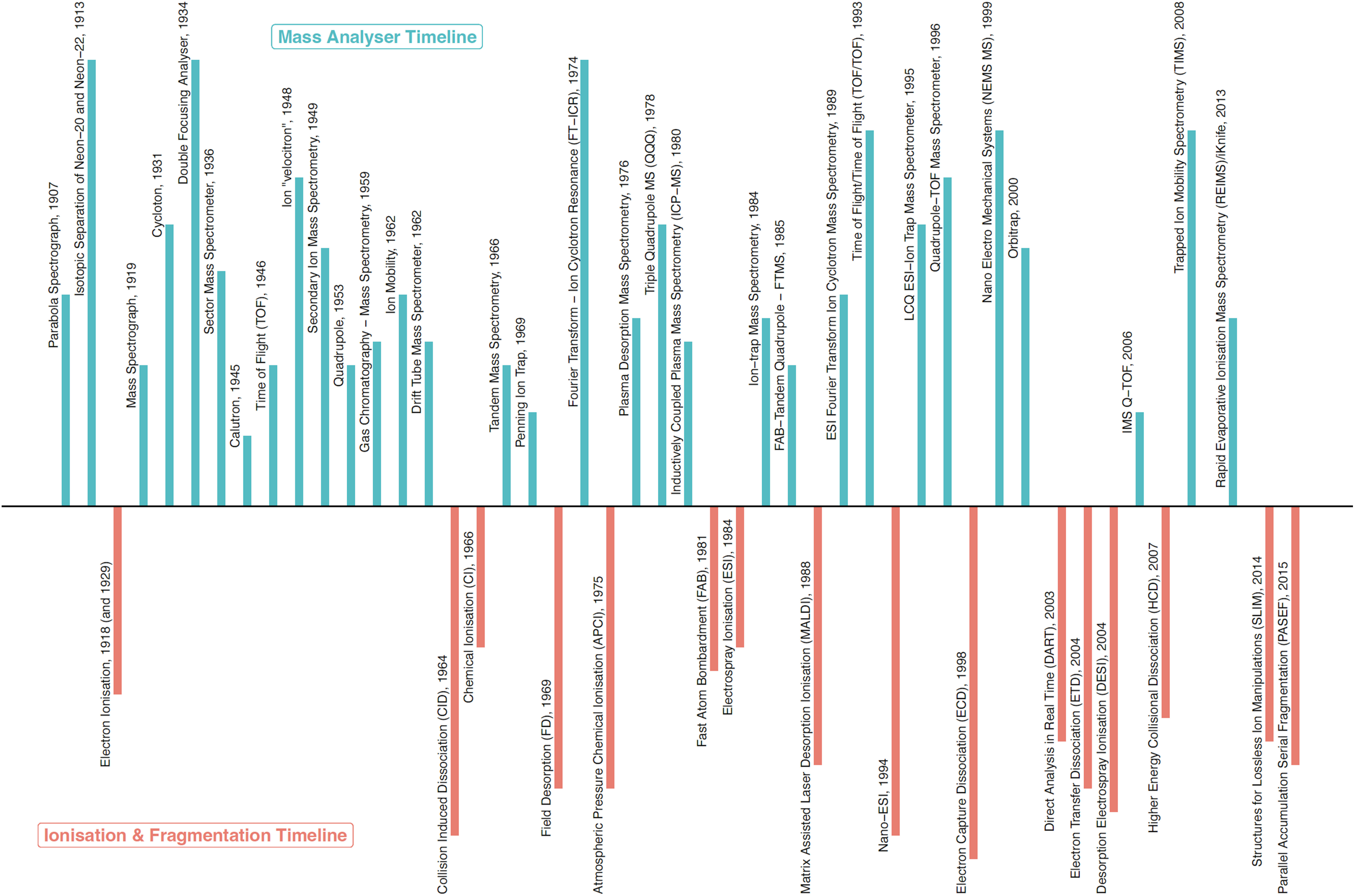
Fig. 1. Timeline of mass spectrometry development.

Mass spectrometry is not a new, or even recently developed, technology. The first MS instrument, known as the parabola spectrograph, was reported in 1907.^1)^ Both mass analysers and ionisation and fragmentation techniques have developed considerably since that time. An outline of the major milestones in MS is shown in Fig. 1.

Originally mass spectrometers were used primarily in physics, to determine the atomic weights of elements for example, or in the discovery of stable isotopes.^1)^ Today however, MS is chiefly used in chemistry and bioscience, to identify and quantify compounds in a particular sample, and to determine the structure of molecules. Due to its sensitivity, robustness and reproducibility, MS has been integral to the continuing advances in chemistry, biochemistry, molecular biology and associated fields in the last 20–30 years.

One of the factors of most interest to the modern mass spectrometrist is the limit of detection (LoD). This can be defined as the lowest amount of a substance that can reliably distinguished from a blank sample.^2)^ Similarly, the limit of quantitation (LoQ) is the lowest concentration at which the analyte can not only be reliably detected, but at which some predefined goals for imprecision and error in the data are met.^2)^

The exact definition of LoD and LoQ are often the subject of much debate. However, they are one of the ways the performance of mass spectrometer is judged. Papers describing new or improved analytical methods, or new instruments are almost required to report these measurements to get published, despite the fact there is no standard agreed, consistent way of determining these values, and considerable discussion on their usefulness.^3)^ Nevertheless, an idea of how sensitive a particular instrument is and how detection limits might improve in future is clearly of some interest/use to analysts and industry.

Manufacturers of mass spectrometers, or indeed any analytical equipment, will understandably want to present their instruments in the best possible light. To this end industry LoDs are often presented that were obtained from a very pure standard, in a very clean matrix, on a very new instrument. These conditions do not represent conditions of most samples run on a MS in laboratories, so do not tell the whole story. It is thus important when discussing how LoDs have advanced over time to include data from research where a LoD was needed but where it was not the sole aim of the work.

The advances in MS limits of detection can be likened to Moore’s law (an observation originally made with only four data points) which states that the number of transistors on silicon chips roughly doubles every 24 months.^4)^

Reports that Moore’s law will soon reach its limit have, to date, proved unfounded but at some point, physical limitations will impede progress as the space between transistors on a chip approach sub-atomic distance. Likewise it would be interesting to ascertain if there is a “law” of MS and thus estimate if the technology is likely to continuously deliver better detection limits and when, if ever, we might reach single molecule detection.^5)^ In this paper we draw on historical data from the literature to assess how MS LoDs have changed over time and investigate the question—is there a Moore’s law of Mass Spectrometry?

## EXPERIMENTAL

### Industry data

The major manufacturers of mass spectrometers worldwide were contacted by e-mail and asked if they had any historical data on LoD that they would be willing share. Most replied stating they did not have such data, or that if they did, it was (not unreasonably) commercially sensitive. One company (SCIEX) kindly provided a link to an online article published by a SCIEX scientist using historical data from the company.^6)^

**Figure 2:**
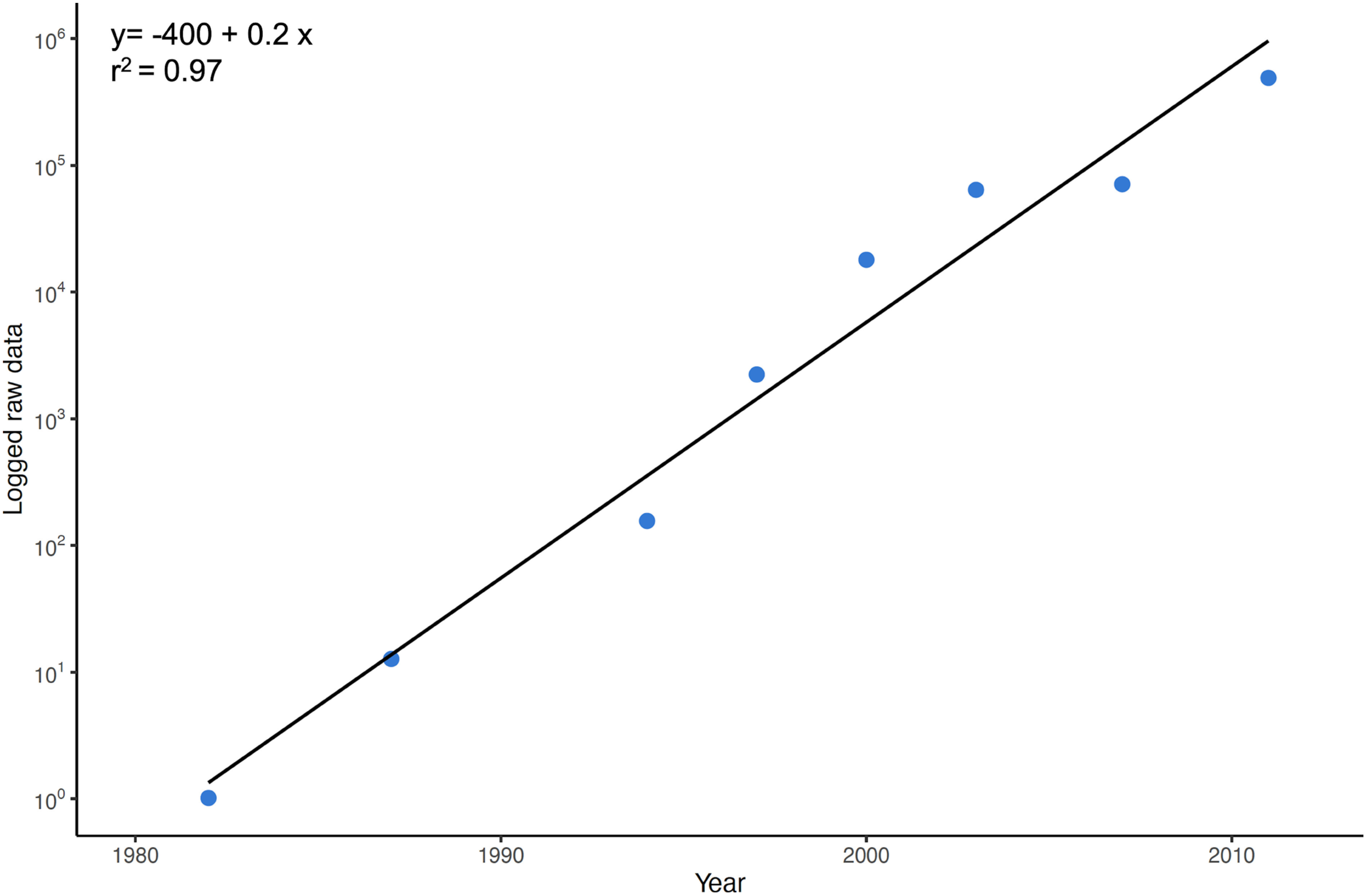
Fig. 2. The improvement of SCIEX MS resolution from 1982 to 2012.

The SCIEX data had already been transformed and normalised when it was published, and it was provided with no units. The company were not able to provide the raw figures so the data were extracted from a figure in the article^6)^ using PlotDigitiser software.^7)^ Said data was then plotted using R statistical software (version 4.1.0) with the ggplot2 package.^8)^ This graph was then used to assess whether the trend in LoD improvement from the industry data resembled Moore’s Law. The graph is shown in Fig. 2, with the data obtained using PlotDigitiser in the supplementary information.

### Literature data

**Figure 3:**
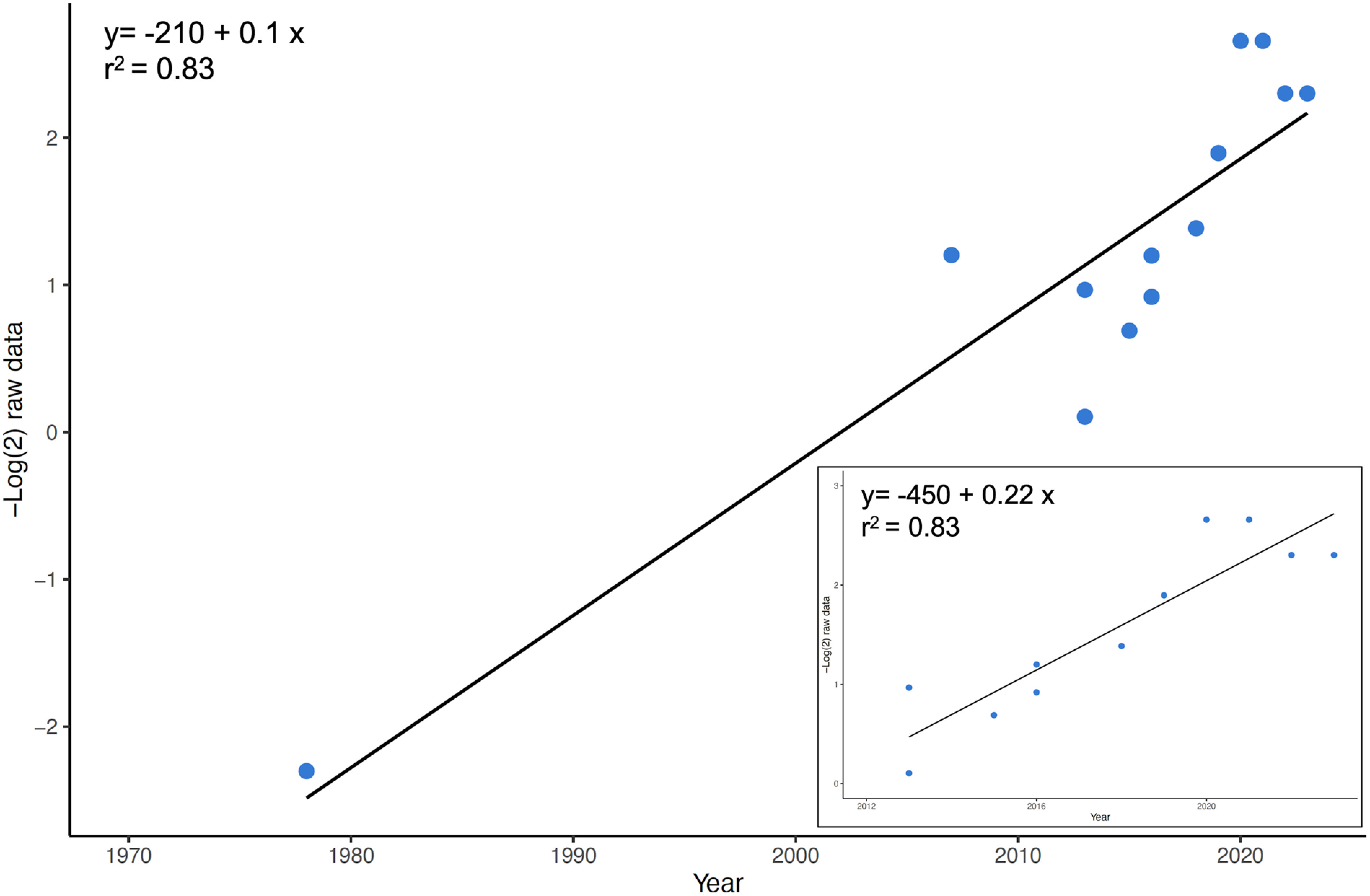
Fig. 3. Limit of detection for MS plotted logarithmically against the date of publication for glycine (1979–2022). Insert shows data from 2012–2023.

**Figure 4:**
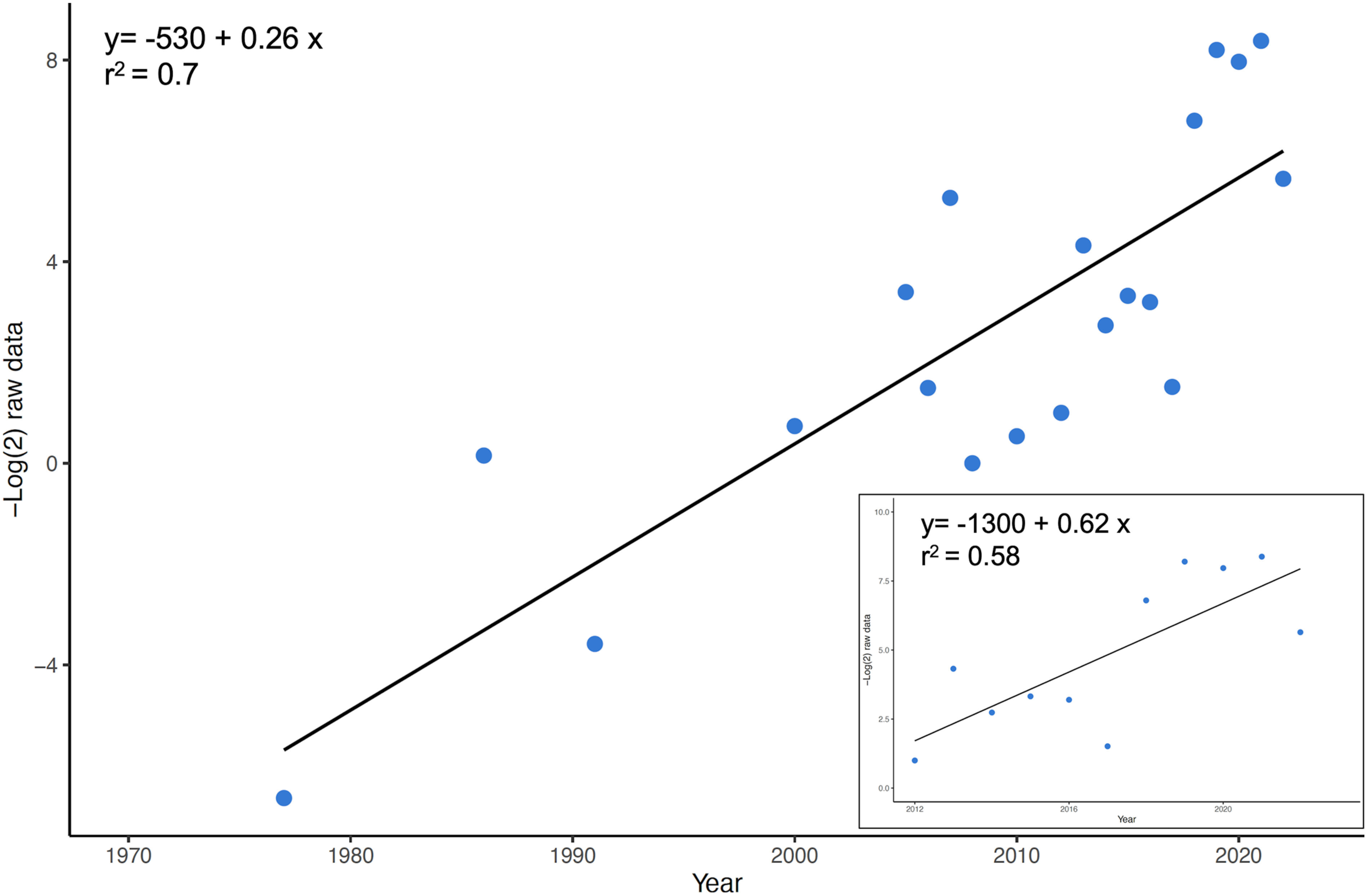
Fig. 4. Limit of detection for MS plotted logarithmically against the date of publication for DDT.

The LoDs for two compounds, glycine (molecular weight 75.07 g/mol) and dichlorodiphenyltrichloroethane (DDT) (molecular weight 353.5 g/mol) were taken from multiple published articles spanning a time period of 45 years. The LoD in the majority of articles was listed as the mass required to produce a signal to noise ratio of 3 : 1. The log of the LoDs were plotted against the article’s year of publication, using R, to assess whether the trend in improvement resembles Moore’s Law. This data is shown in Figs. 3 and 4.

## RESULTS AND DISCUSSION

The results of the industry data are shown in Fig. 2. Each data point on the original chart represents the absolute sensitivity on high-flow LC/MS instruments (at flow rates of ∼1 mL per minute) using SCIEX historical data, beginning in 1982.

Figure 2 shows that, over the 30 years of data, the sensitivity of SCIEX mass spectrometers increased by nearly a factor of one million. The points are plotted on a logarithmic vertical axis, normalised to the data point at 1982, when SCIEX introduced its first LC/MS interface. If we assume the data is on a −log10 scale, then the slope of 0.2 is just above what would be expected from Moore’s Law. Improvement of a factor of one million over 30 years is a greater rate of improvement than Moore’s law.

The raw data or the units were not provided, so it is impossible to back calculate the original concentrations. However, the SCIEX report notes that in 1982, it typically required an injection of nanogram amounts of a compound to obtain good signal-to-noise.^6)^ Today, some workers report they can quantitatively measure injected amounts of drugs or metabolites at the sub-femtogram level. This represents ∼0.001 part-per-trillion (ppt) concentration in the 1 mL sample. If we assume the first data point in Fig. 2 is 1 ng per mL (or 1 μg per L) then a millionfold improvement would indeed be in the femtogram per mL (or picograms per litre range). This fits with the improvement of a million-fold over the stated time period.

The SCIEX data^6)^ did not include any information on how it was gathered (other than the flow rate) or what substance was analysed, or what matrix is was in, or how the signal to noise was calculated. It also comes only from SCIEX instruments and thus may not reflect the rate of advancement of the industry as a whole.

One problem with comparing the sensitivity specifications over time is that a range of test compounds have been used over the years. Today octafluoronaphthalene (OFN) is used but before this it was common to use hexachlorobenzene. Drawing parallels is thus difficult. It may be better to concentrate on how sensitivity specs have changed over the last 15–25 years when OFN has been the norm. Replotting the data in Fig. 2 to include only data from 1995 to 2012 gives a slope of Y=−290+0.15x (with a r^2^ value of 0.91) which is about the same rate of increase as Moore’s law.

Impressive as the SCIEX data is, it must be kept in mind that it was likely achieved using a very clean sample, of a single pure compound on the most modern instrument available. It does not necessarily reflect the conditions of everyday labs where samples may have been extracted from a complex matrix, be part of a complex mixture of compounds, and/or run on an instrument that is several years old, running many other sample types. To allow a comparison of data from across different laboratories and spectrometers LoD data from the same compounds over an extended period of time was needed. It was thus helpful to look at historic data from the literature.

Reported LoDs for glycine over time is shown is Fig. 3. Data in Fig. 3 is taken from the references.^9–20)^ Since the range of the data was not as large as the SCIEX data a Log2 scale was used rather than Log10. The raw data is given in the supplementary information.

The rate of improvement in LoDs for glycine is exponential, however the gradient of the trend line is 0.1 which is below what is required to satisfy Moore’s Law. The data in Fig. 3 may, however, be overly influenced by the point in 1978. There is much steeper rate of improvement between 2012 and 2022. If only the data over the last ten years is plotted, there is a much steeper rate of advancement with a slope of 0.2 (shown in the insert of Fig. 3). This is still below the slope of 0.5 we would expect to see with Moore’s law at a Log2 scale.

The reported LoD for DDT over time is shown in Fig. 4. Data in Fig. 4 is taken from the references.^21–40)^ The raw data is again given in the supplementary information. The data is again presented at the Log2 scale.

While there is more data for DDT than glycine it is more variable. The data is not overly influenced by the earlier data points although there are more data after the year 2000. The rate of increase is again exponential with a gradient of 0.26 which is roughly equal to that of glycine. If the last ten years of data is plotted the slope of the line is 0.62 which is higher than that of Moore’s law, with the caveat that the data is much more spread out with an r^2^ of 0.56.

There is significant contrast in the rate of improvement between the LoDs taken from articles in the literature and the information provided by industry, with the rate of improvement in industry considerably higher than that from the literature. Additionally, although the overall trend in LoD is upward, reported detection limits from the literature do not continually improve each year as the industry data does. Instead, they fluctuate. This is likely the result of data being obtained from complex samples run on different instruments; a triple quadrupole instrument compared to a single quadrupole for example.

It should also be noted that the rate of improvement in the LoD is not necessarily representative of the improvement of resolution for all of mass spectrometry as a technique. The exercise would have to be repeated for molecules of varying size and complexity to make the conclusion more certain. The sparse number of available articles from prior to 2000 also calls into question whether the determined rate of improvement is applicable to earlier points in time. The LoDs are also organised by year of publication and not age of mass spectrometer used in the method. Changes to the LoD from year to year can sometimes be minimal or non-existent because the MS used in a laboratory will often remain the same for several years.

Another issue is that each publication sourced to form Figs. 3 and 4 is different, there are potential variables in the methods other than the mass spectrometer used that could affect the LoD. Such variables include sample preparation, sample type and data processing. It is becoming common practice now to use a statistical approach to identifying the LoD for a particular instrument. In such a regime the resolution of a detector scales with the number of measurements across a peak. Assuming the peak associated with a desired measurement follows a Poisson distribution then there will be a minimum number of measurements before a peak can be isolated from the background measurements as this creates the limit for any measurement.

In summary, while there is variation between compounds, the LoD for MS appears to be increasingly exponentially and is just below that of Moore’s law over the last ten years. One study has recently estimated increases in LoD would mean that single molecule detection could be possible in “routine” mass spectrometry in 2032.^5)^

It should perhaps be noted that Moore’s law is not actually a law, but rather an observation/prediction made many years ago. It has since become a self-fulfilling prophesy and a key target for a large, motivated, and well-funded industry. In contrast, while analysts are obviously keen on lowering LoD, which drives industry to improve, there is no similar, formal rate of improvement goal driving the mass spectrometry industry to lower the limit of detection as Moore’s law does in computing.

Despite ongoing improvements in ion sources, optics, and vacuum systems advances in mass spectrometry will become harder as the industry nears single molecule/ion detection. Just as the computer industry has started to use alternative to Moore’s law—such as number of GPU processing tasks, MS may shift to developing faster, more selective, and more economical techniques. There will be significant challenges to this. Nevertheless, while the future of mass spectrometry is not certain, it is certain to remain a crucial research tool and reported limits of detection are certain to keep improving.

## CONCLUSION

Increases in the LoD of mass spectrometry have a trend similar to (but just below) that of Moore’s law, however there in no universal metric to use when comparing MS LoDs and significant variation between instruments and methods. This makes direct comparison difficult. As the needs of the analytical community continue to increase, it is likely that further and improved approaches to generating, sampling, and detecting ions by mass spectrometry will be developed. Both computers and mass spectrometers have developed from room sized instrument that could only be operated by specialists, to much more versatile systems that can be used by even non-specialists to generate useful data. While it is not yet possible to have mass spectrometers in the home, their wide use for a huge range of scientific applications will likely keep driving improvements in LoD for the foreseeable future.

## Conflicts of Interest

The authors declare that they have no known competing financial interests or personal relationships that could have appeared to influence the work reported in this paper.

## Notes

Mass Spectrom (Tokyo) 2023; 12(1): A0118
