# Supplementary material for: Rate of Advancement of Detection Limits in Mass Spectrometry: Is there a Moore’s Law of Mass Spec?
Source: Mass Spectrom (Tokyo). 2023 Apr 6;12(1):A0118. doi: 10.5702/massspectrometry.A0118 (PMC10209656; doi:10.5702/massspectrometry.A0118)
Supplement: Supplementary Data [file massspectrometry-12-1-A0118_s001.pdf]

Supplementary Material

Rate of Advancement of Detection Limits in Mass Spectrometry: Is there a Moore's Law of Mass Spec?

Mark Beattie and Oliver A.H. Jones*

Australian Centre for Research on Separation Science (ACROSS), School of Science, RMIT University, GPO Box 2476, Melbourne, VIC 3001, Australia

* Author to whom correspondence may be addressed

Phone: +61 (0) 3 9925 2632
Fax: +61 (0) 3 9925 3747
E-mail: oliver.jones@rmit.edu.au

Content

Table S1. Raw data behind figure 2 of main manuscript
Table S2. Raw data behind figure 3 of main manuscript
Table S3. Raw data behind figure 4 of main manuscript
References

Table S1. Raw data behind figure 2 of main manuscript.

NB: All data extracted from the graph in Thompson [1] using PlotDigitiser [2] so some errors could be present. No raw data or units or data processing information were included in Thompson.[1] The data should therefore be treated as estimated values.

Year	Raw data
1982	1.00
1987	12.71
1994	156.27
1997	2237.14
2000	17997.61
2003	64174.42
2007	71045.04
2011	490679.08

Table S2. Raw data behind figure 3 of main manuscript

**Year**	**Limit of detection (µg/L)**	**-Log2 (LoD)**	**Reference**
1978	10	-3.32193	3)
2007	0.3	1.736966	4)
2013	0.9	0.15	5)
2015	0.5	1.00	6)
2016	0.3	1.74	7)
2016	0.4	1.32	8)
2018	0.25	2.00	9)
2019	0.15	2.74	10)
2020	0.07	3.84	11)
2021	0.07	3.84	12)
2022	0.1	3.32	13)
2023	0.1	3.32	14)

Table S3. Raw data behind figure 4 of main manuscript

Year	Limit of detection (µg/L)	-Log2 (LoD)	Reference
1977	100	-6.64	[15)]
1986	0.9	0.15	[16)]
1991	12	-3.58	[17)]
2000	0.6	0.74	[18)]
2005	0.10	3.40	[19)]
2006	0.36	1.49	[20)]
2007	0.03	5.27	[21)]
2008	1	0.00	[22)]
2010	0.69	0.54	[23)]
2012	0.5	1	[24)]
2013	0.05	4.32	[25)]
2014	0.15	2.74	[26)]
2015	0.1	3.32	[27)]
2016	0.109	3.20	[28)]
2017	0.35	1.51	[29)]
2018	0.009	6.80	[30)]
2019	0.0034	8.20	[31)]
2020	0.004	7.97	[32)]
2021	0.003	8.38	[33)]
2022	0.02	5.64	[34)]

REFERENCES

1) B. Thomson. Driving High Sensitivity in Biomolecular MS. *Genett Eng. Biotechnol. News* 32, 2012.
2) PlotDigitizer - https://plotdigitizer.com.
3) A. Benninghoven, W. K. Sichtermann. Detection, identification, and structural investigation of biologically important compounds by secondary ion mass spectrometry. *Anal.Chem.* 50: 1180-1184, 1978.
4) H. Yoshida, T. Mizukoshi, K. Hirayama, H. Miyano. Comprehensive Analytical Method for the Determination of Hydrophilic Metabolites by High-Performance Liquid Chromatography and Mass Spectrometry. *J. Agric. Food Chem.* 55: 551-560, 2007.
5) J. Cui, J. Zhang, X. Zhu, F. Bai, Y. Feng, W. Guan, Q. Cui. separation and quantification of water-soluble cellular metabolites in *Clostridium thermocellum* using liquid chromatography-isotope dilution tandem mass spectrometry. *Anal. Lett.* 46: 2767-2786, 2013.
6) A. D. Troise, A. Fiore, G. Roviello, S. M. Monti, V. Fogliano. Simultaneous quantification of amino acids and Amadori products in foods through ion-pairing liquid chromatography–high-resolution mass spectrometry. *Amino Acids* 47: 111-124, 2015.
7) K. Horňák, H. Schmidheiny, J. Pernthaler. High-throughput determination of dissolved free amino acids in unconcentrated freshwater by ion-pairing liquid chromatography and mass spectrometry. *J. Chromatogr. A* 1440: 85-93, 2016.
8) B. Yin, T. Li, S. Zhang, Z. Li, P. He. Sensitive Analysis of 33 Free Amino Acids in Serum, Milk, and Muscle by Ultra-High Performance Liquid Chromatography-Quadrupole-Orbitrap High Resolution Mass Spectrometry. *Food Anal. Methods* 9: 2814-2823, 2016.
9) L. Konieczna, M. Pyszka, M. Okońska, M. Niedźwiecki, T. Bączek. Bioanalysis of underivatized amino acids in non-invasive exhaled breath condensate samples using liquid chromatography coupled with tandem mass spectrometry. *J. Chromatogr. A* 1542: 72-81, 2018.
10) R. M. Kazan, H. A. Seddik, Z. M. Marstani, M. M. Elsutohy, N. G. Yasri. Determination of amino acids content in tea species using liquid chromatography via pre-column fluorescence derivatization. *Microchem. J.* 150: 104103, 2019.
11) X. N. Wang, J. Q. Liu, Z. Q. Shi, F. Y. Sun, L. F. Liu, G. Z. Xin. Orthogonal label and label-free dual pretreatment for targeted profiling of neurotransmitters in enteric nervous system. *Anal Chim Acta* 1139: 68-78, 2020.
12) E. M. Gargano, S. Sell, S. Langhoff, C. U. Schmidt, S. Wierlacher. Development and validation of a method for simultaneous analysis of hair underivatized amino acids and damage biomarkers, using liquid chromatography-tandem mass spectrometry. *Talanta* 233: 122584, 2021.
13) S. Chen, Y. Fu, X. Bian, M. Zhao, Y. Zuo, Y. Ge, Y. Xiao, J. Xiao, N. Li, J.-L. Wu. Investigation and dynamic profiling of oligopeptides, free amino acids and derivatives during Pu-erh tea fermentation by ultra-high performance liquid chromatography tandem mass spectrometry. *Food Chem.* 371: 131176, 2022.
14) N. K. N. Bùi, S. Selberg, K. Herodes, I. Leito. Coumarin-based derivatization reagent for LC-MS analysis of amino acids. *Talanta* 252: 123730, 2023.

15) G. D. Veith, D. W. Kuehl, F. A. Puglisi, G. E. Glass, J. G. Eaton. Residues of PCB's and DDT in the western Lake Superior ecosystem. *Arch. Environ. Contam. Toxicol.* 5: 487-499, 1977.
16) A. L. Alford-Stevens, T. A. Bellar, J. W. Eichelberger, W. L. Budde. Accuracy and precision of determinations of chlorinated pesticides and polychlorinated biphenyls with automated interpretation of mass spectrometric data. *Anal Chem* 58: 2022-2029, 1986.
17) H. Behzadi, R. A. Lalancette. Extending the use of USEPA method 1625 for the analysis of 4,4′-DDT, 4,4′-DDD, and 4,4′-DDE. *Microchem. J.* 44: 122-129, 1991.
18) B.-H. Hwang, M.-R. Lee. Solid-phase microextraction for organochlorine pesticide residues analysis in Chinese herbal formulations. *J. Chromatogr. A* 898: 245-256, 2000.
19) J. L. M. Vidal, F. J. A. Liébanas, M. J. G. Rodríguez, A. G. Frenich, J. L. F. Moreno. Validation of a gas chromatography/triple quadrupole mass spectrometry based method for the quantification of pesticides in food commodities. *Rapid Commun. Mass. Spectrom.* 20: 365-375, 2006.
20) A. Garrido Frenich, J. L. Martínez Vidal, A. D. Cruz Sicilia, M. J. González Rodríguez, P. Plaza Bolaños. Multiresidue analysis of organochlorine and organophosphorus pesticides in muscle of chicken, pork and lamb by gas chromatography–triple quadrupole mass spectrometry. *Anal. Chim. Acta* 558: 42-52, 2006.
21) A. Derouiche, M. R. Driss, J.-P. Morizur, M.-H. Taphanel. Simultaneous analysis of polychlorinated biphenyls and organochlorine pesticides in water by headspace solid-phase microextraction with gas chromatography–tandem mass spectrometry. *J. Chromatogr. A* 1138: 231-243, 2007.
22) K. Banerjee, S. H. Patil, S. Dasgupta, D. P. Oulkar, S. B. Patil, R. Savant, P. G. Adsule. Optimization of separation and detection conditions for the multiresidue analysis of pesticides in grapes by comprehensive two-dimensional gas chromatography–time-of-flight mass spectrometry. *J. Chromatogr. A* 1190: 350-357, 2008.
23) M. Barriada-Pereira, P. Serôdio, M. J. González-Castro, J. M. F. Nogueira. Determination of organochlorine pesticides in vegetable matrices by stir bar sorptive extraction with liquid desorption and large volume injection-gas chromatography–mass spectrometry towards compliance with European Union directives. *J. Chromatogr. A* 1217: 119-126, 2010.
24) G. Salquèbre, C. Schummer, M. Millet, O. Briand, B. M. R. Appenzeller. Multi-class pesticide analysis in human hair by gas chromatography tandem (triple quadrupole) mass spectrometry with solid phase microextraction and liquid injection. *Anal. Chim. Acta* 710: 65-74, 2012.
25) M. L. Martins, F. F. Donato, O. D. Prestes, M. B. Adaime, R. Zanella. Determination of pesticide residues and related compounds in water and industrial effluent by solid-phase extraction and gas chromatography coupled to triple quadrupole mass spectrometry. *Anal. Bioanal. Chem.* 405: 7697-7709, 2013.
26) P. Deme, V. V. R. Upadhyayula. Ultra performance liquid chromatography atmospheric pressure photoionization high resolution mass spectrometric method for determination of multiclass pesticide residues in grape and mango juices. *Food Chem.* 173: 1142-1149, 2014.
27) Y. Jiang, M. Zhong, Y. Gou, W. Peng, J. Zhou, P. Wu, Y. Ma. Development of a more specific and accurate multiple reaction monitoring method based on GC–

EI/MS/MS for simultaneously monitoring and determining 34 kinds of pesticides in Qianjinzhidai pills. *J. Chromatogr. B* 983-984: 47-54, 2015.
28) M. G. Pintado-Herrera, E. González-Mazo, P. A. Lara-Martín. In-cell clean-up pressurized liquid extraction and gas chromatography–tandem mass spectrometry determination of hydrophobic persistent and emerging organic pollutants in coastal sediments. *J. Chromatogr. A* 1429: 107-118, 2016.
29) M. Sajid, C. Basheer, M. Daud, A. Alsharaa. Evaluation of layered double hydroxide/graphene hybrid as a sorbent in membrane-protected stir-bar supported micro-solid-phase extraction for determination of organochlorine pesticides in urine samples. *J. Chromatogr. A* 1489: 1-8, 2017.
30) A. Szarka, D. Turková, S. Hrouzková. Dispersive liquid-liquid microextraction followed by gas chromatography–mass spectrometry for the determination of pesticide residues in nutraceutical drops. *J. Chromatogr. A* 1570: 126-134, 2018.
31) T. G. Schwanz, C. K. Carpilovsky, G. C. C. Weis, I. H. Costabeber. Validation of a multi-residue method and estimation of measurement uncertainty of pesticides in drinking water using gas chromatography–mass spectrometry and liquid chromatography–tandem mass spectrometry. *J. Chromatogr. A* 1585: 10-18, 2019.
32) J. E. Lee, H. B. Oh, H. Im, S. B. Han, K. H. Kim. Multiresidue analysis of 85 persistent organic pollutants in small human serum samples by modified QuEChERS preparation with different ionization sources in mass spectrometry. *J. Chromatogr. A* 1623: 461170, 2020.
33) J. S. Hinz, L. Morés, E. Carasek. Exploring the use of cork pellets in bar adsorptive microextraction for the determination of organochloride pesticides in water samples with gas chromatography/electron capture detection quantification. *J. Chromatogr. A* 1645: 462099, 2021.
34) X. Sun, Z. Fu, T. Jiang, F. Ning, Y. Cheng, T. Fu, M. Zhu, H. Zhang, M. Zhang, P. Hu. Application of β-Cyclodextrin metal-organic framework/titanium dioxide hybrid nanocomposite as dispersive solid-phase extraction adsorbent to organochlorine pesticide residues in honey samples. *J. Chromatogr. A* 1663: 462750, 2022.
